# Non-diabetic Euglycemic Ketoacidosis in an Adult Patient With Spinal Muscular Atrophy Type II

**DOI:** 10.7759/cureus.45666

**Published:** 2023-09-21

**Authors:** George Khludenev, Elise Le Cam, Bujji B Ainapurapu

**Affiliations:** 1 Internal Medicine, University of Arizona College of Medicine - Tucson, Tucson, USA

**Keywords:** glucose metabolism, intractable nausea, metabolism disorder, starvation ketoacidosis, spinal muscular atrophy (sma)

## Abstract

Spinal muscular atrophy (SMA) is a rare neuromuscular disease that develops as a result of the degeneration of the anterior horn cells in the spinal cord and lower brainstem motor nuclei, resulting in progressive muscle weakness and atrophy. While the initial presentation of this disease involves diffuse muscular atrophy at an early age, patients with an established diagnosis and later-stage disease often present with gastrointestinal symptoms related to metabolic imbalances. Here, we examine the case of an adult patient with SMA type II who presented with complaints of 12 hours of intractable nausea and vomiting. The patient was found to be in euglycemic ketoacidosis (EKA), an uncommon, but not unheard of, metabolic derangement in SMA patients with severely decreased muscle mass.

## Introduction

Spinal muscular atrophy (SMA) is a rare, well-characterized neuromuscular disease defined by progressive muscular atrophy resulting from the degeneration of motor neurons. The pathology of this illness is caused by an inherited, autosomal recessive mutation of the survival motor neuron 1 (*SMN1*) gene on chromosome 5q13.2 [1]. Moreover, SMA is classified into seven phenotypes based on varied clinical presentations whose variance seems to be related to the function of the survival motor neuron 2 (*SMN2*) gene [1]. Euglycemic ketoacidosis (EKA) is an uncommon but well-described metabolic derangement accounting for 2.6%-3.2% of admissions for diabetic ketoacidosis (DKA) [2]. This diagnosis is defined as the triad of high anion gap metabolic acidosis, elevated plasma ketones, and normal blood glucose [3]. While this metabolic derangement is most frequently associated with either type I or type II diabetes mellitus, other distinct pathologic entities may result in EKA, including starvation, pregnancy, heavy alcohol consumption, and inborn errors of metabolism [4]. Patients with progressive SMA are often at greater risk of ketoacidosis due to a deficiency of muscle mass, which lends itself to a greater propensity for ketogenesis and ketoacidosis.

## Case presentation

A 23-year-old male was admitted to the general medical floor from the emergency department with complaints of approximately 12 hours of severe nausea and vomiting with complete intolerance of any food or water by mouth. The patient reported one episode of clear, nonbilious vomit directly after attempting to drink water but denied any evidence of hematemesis. The patient’s past medical history was significant for spinal muscular atrophy type II diagnosed in infancy with resultant failure to thrive, scoliosis with complete surgical spinal fusion, and asthma. The patient reported taking risdiplam 6.6 mL once daily for his SMA type II as well as fluticasone, albuterol, and ipratropium inhalers as needed for his asthma. Of note, the patient denied any significant alcohol or tobacco use but endorsed daily medical marijuana use for appetite stimulation and multimodal pain control.

**Figure 1:**
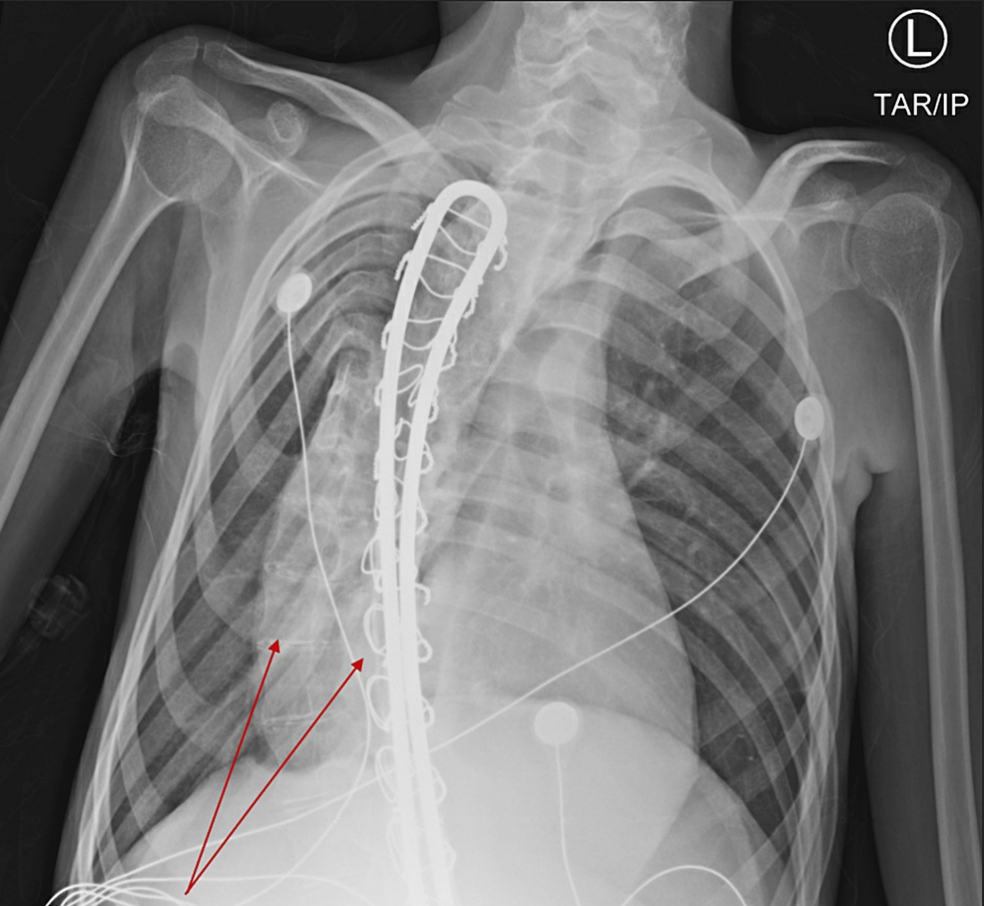
Chest X-Ray Demonstrating Severe Scoliosis and Previous Spinal Fusion (Arrows)

On presentation, laboratory blood work was significant for high anion gap metabolic acidosis with respiratory compensation. Venous blood gas measurements on admission demonstrated a low-normal pH of 7.35, reduced serum bicarbonate (HCO_3_-) of 15 mmol/L, and partial pressure of CO_2_ (pCO_2_) of 35 mmHg, suggesting a very mild, compensated metabolic acidosis (Table 1). Otherwise, initial laboratory findings were significant for a leukocytosis, a high anion gap, and normal blood glucose (Table 2), a urinalysis positive for urinary ketones, and an elevated beta-hydroxybutyrate blood serum level of 41 mg/dL (reference range: <2.8 mg/dL). Blood serum aspirin and salicylate levels were both undetectable on admission. An infectious workup was initiated with blood cultures drawn and a gastrointestinal viral panel sent for analysis. Plain-film chest radiography on admission redemonstrated known severe scoliosis and prior spinal fusion but showed no evidence of an acute infectious process (Figure 1).

**Table 1:** Venous Blood Gasses on Admission pCO_2_: partial pressure of CO_2_, pO_2_: partial pressure of O_2_, HCO_3 _^-^: bicarbonate

Venous blood gasses	Values	Reference ranges
pH	7.35	7.35-7.45
pCO_2_	34 mmHg	41-51 mmHg
pO_2_	62 mmHg	30-45 mmHg
HCO_3_-	15 mmol/L	22-28 mmol/L

**Table 2:** Pertinent Laboratory Tests Throughout Admission

Pertinent laboratory tests	Values on admission	12 hours after admission	24 hours after admission	36 hours after admission	Reference ranges
White blood cell count	26,800 cells/uL	23,800 cells/uL	13,800 cells/uL	9,800 cells/uL	4,000-11,000 cells/uL
Anion gap	23	19	14	10	4-16
Lactic acid	0.8 mmol/L	0.6 mmol/L	None measured	None measured	0.5-2 mmol/L
Glucose	71 mg/dL	112 mg/dL	95 mg/dL	96 mg/dL	70-106 mg/dL
Serum bicarbonate	15 mmol/L	13 mmol/L	19 mmol/L	24 mmol/L	19-31 mmol/L
Serum sodium	138 mmol/L	132 mmol/L	139 mmol/L	140 mmol/L	134-147 mmol/L
Serum potassium	4 mmol/L	2.8 mmol/L	3.9 mmol/L	4.2 mmol/L	3.6-5.3 mmol/L

Initially, the patient’s presentation suggested a mixed starvation ketoacidosis and contraction alkalosis with respiratory compensation secondary to marijuana hyperemesis. Infectious gastroenteritis was also considered as an alternative diagnosis given the patient’s markedly elevated white blood cell count. The patient was given a bolus of 1.5 L of crystalloid fluid and started on a dextrose 10% infusion at a rate of 50 cc/hour for volume resuscitation. Given that this patient’s body weight at presentation was 31.8 kg, initial fluids administered as smaller boluses of 500 cc corresponding to approximately 15 mL/kg of fluid resuscitation were deemed adequate during the initial phase of illness in DKA.

On the second day of hospitalization, the patient’s repeat laboratory results showed an improving anion gap but worsening bicarbonate levels as well as new hypokalemia and hyponatremia (Table 2) likely from increased insulin activity, over-resuscitation with fluids, and volume expansion. The patient was able to tolerate good PO intake but continued to complain of moderate nausea and abdominal pain. The infectious workup had yielded no definitive evidence of gastrointestinal infection, and abstinence from marijuana did not relieve the patient’s symptoms, so the patient was continued on a dextrose 10% infusion at 50 cc/hour under suspicion of resolving EKA diagnosis. Following a 10% dextrose infusion for approximately 30 hours total, repeat blood investigations on the third day of admission showed complete resolution of leukocytosis, normalization of serum electrolytes, and a closed serum anion gap (Table 2). Along with the resolution of metabolic derangements, the patient was able to maintain adequate oral fluid intake by this point, and a net total of 1.5 L of 10% dextrose was ultimately administered in addition to the initial 1.5 L of crystalloid fluid boluses.

Upon reviewing old records, the patient’s previous pediatric admissions had documented euglycemic ketoacidosis secondary to severely decreased muscle mass and in conjunction with elevated blood serum beta-hydroxybutyrate levels. He also underwent a detailed metabolic workup as an outpatient, the results of which unfortunately could not be obtained. The patient was subsequently started on L-carnitine and discharged home in stable condition. He later followed up with his childhood pediatrician for routine post-hospitalization care.

## Discussion

While patients with SMA are known to have metabolic derangements resulting from impaired glucose, protein, and lipid metabolism, non-diabetic EKA is rarely considered in light of more common etiologies of intractable nausea and vomiting. Nonetheless, limited case reports do exist detailing similar presentations, and there is value in contributing to further evidence of this pathology in this population [5,6]. It is important for providers seeing these patients to be aware of the pathophysiology and laboratory workup of EKA as well as the available therapeutic interventions for this condition.

Although not well described in the literature, some limited studies exist that detail the fatty acid oxidation abnormalities in patients with SMA [7]. Currently, it is thought that patients with SMA may develop EKA as a result of an imbalance between glucose and ketone metabolism. In the normal physiological context, glucose is stored as glycogen in both the liver and skeletal muscles. When serum glucose and hepatic glycogen stores are depleted, the liver will attempt to generate new glucose through gluconeogenesis by relying on glycerol from stored fat and eventually amino acids from the skeletal muscle catabolism [8]. If gluconeogenesis is still insufficient to meet the body’s metabolic demands, the liver begins ketogenesis to continue fueling activity. Thus, hepatic ketogenesis is promoted when glucose stores are depleted and gluconeogenesis is insufficient to meet metabolic demand [9]. Importantly, the peripheral skeletal muscles are involved in both the supply of amino acids for gluconeogenesis and the uptake of ketones when the body enters ketosis [8]. In patients who have severely decreased musculature, such as those with SMA, starvation will both accelerate the onset of hepatic ketogenesis given the reduced availability of glycogen and decrease the uptake of these ketones resulting in a more rapid onset of serum ketoacidosis. Therefore, in patients with later-stage spinal muscular atrophy, the threshold for ketoacidosis during periods of starvation is much lower than that in normal metabolism, and so, these patients ought to be closely monitored for metabolic derangements when presenting with abdominal symptoms and poor oral intake.

When any patient presents with signs of nausea and vomiting, initial evaluation should screen for common etiologies with tests such as a complete blood count with differential, a complete metabolic panel, a gastrointestinal infectious panel, and others. In the context of an elevated anion gap, further tests including blood plasma ketones, lactic acid, salicylate level, and alcohol level should also be considered to assist in ruling out common causes of this finding. In a patient with SMA, serum and urinary ketones should be ordered to rule out starvation ketoacidosis and help identify euglycemic ketoacidosis if present. Serum levels of free amino acids (i.e., alanine) should also be considered as a lower availability impairs gluconeogenesis, increasing conversion to ketogenesis [10]. Finally, with the initial presentation of high anion gap metabolic acidosis in an SMA patient, urine organic acids, carnitine level, acylcarnitine level, and free fatty acids should be ordered to further evaluate and exclude for potential metabolic defects and inborn errors of metabolism that have historically been described in these patients [11].

Finally, considering very few case reports have been published regarding non-diabetic euglycemic ketoacidosis in SMA patients, treatment remains uncertain at this time. One case report suggested aggressive hydration and volume expansion [5], while another proposed the application of a diet rich in carbohydrates and proteins [6]. Considering our patient’s presentation with intractable nausea and vomiting, fluid resuscitation was the initial focus of management to ensure adequate tissue perfusion and resolution of metabolic abnormalities. Following appropriate fluid resuscitation for his body weight, the patient improved with return of oral intake, and metabolic derangements resolved following the administration of maintenance fluids with dextrose 10% (D10W). D10W was specifically chosen to inhibit ketosis by promoting glycolysis and glycogenesis and therefore restore normal cellular utilization [12]. Although little benefit has been shown from L-carnitine supplementation in SMA patients [13], the decision was made to start the patient on a short course of L-carnitine to potentially inhibit fatty acid oxidation and ketone production. This was prescribed with the intention of hopefully preventing further admissions for the same presentation.

## Conclusions

In conclusion, this case presented the metabolic derangements of a patient with spinal muscular atrophy type II who presented with intractable nausea and vomiting and was found to be in non-diabetic euglycemic ketoacidosis. Although far more common conditions classically cause similar metabolic derangements, the differential diagnoses considered should be broadened and targeted to conditions that are rare but could arise in SMA patients. Our case report helps add to the limited publications available regarding EKA in SMA patients and assists in delineating the pathophysiology, diagnostic workup, and potential treatment plan that should be implemented in order to rapidly resolve metabolic derangements and symptomatology. This case also emphasizes the importance of adult physicians being aware of conditions more commonly encountered in the pediatric population.
